# Assessing the predictive potential of the Holstein bovine sperm methylome for bull fertility using machine learning

**DOI:** 10.1186/s12711-026-01097-y

**Published:** 2026-10-30

**Authors:** A. Asset, V. Costes, C. Fouéré, A. Raja-Ravi-Shankar, C. Le Danvic, F. Jaffrézic, H. Kiefer, D. Makowski

**Affiliations:** 1 Université Paris-Saclay, UVSQ, INRAE, BREED, Jouy-en-Josas, 78350 France; 2 Ecole Nationale Vétérinaire d’Alfort, BREED, Maisons-Alfort, 94700 France; 3 Eliance, 149 rue de Bercy, Paris, 75012 France; 4 Université Paris-Saclay, INRAE, AgroParisTech, GABI, Jouy-en-Josas, France; 5 Université Paris-Saclay/INRAE/ AgroParisTech, UMR MIA Paris-Saclay, Palaiseau, France

## Abstract

**Background:**

Bull fertility is a key parameter in the dairy cattle industry, with significant economic implications. DNA methylation is an epigenetic mark essential for fertility, and recent works have highlighted sperm DNA methylation as a promising source of biomarkers for male fertility, but no operational tool has been developed for predicting bull fertility based on DNA methylation. Here, we leveraged existing methylation data to evaluate the potential of DNA methylation for predicting bull fertility with machine learning models.

**Results:**

Our study was conducted on a cohort of 351 Holstein commercial bulls, classified as subfertile, neutral and fertile based on a field fertility index (non-return rate of the inseminated cows after 56 days post-insemination). This dataset includes the largest cohort assembled for bull fertility prediction from epigenetic markers. We developed classification models to discriminate subfertile and fertile individuals using a subset of predictors derived from reduced representation bisulfite sequencing data obtained on frozen semen. Four machine learning algorithms were compared: Least Absolute Shrinkage and Selection Operator (LASSO), Random Forest, Gradient Boosting, and Neural Networks. To optimize performances of each algorithm and conduct an unbiased assessment of classification accuracy, we used a three-fold cross-validation before final testing on an independent test set. To increase the robustness of the results, model performances were averaged over 20 partitions of the data into training and test sets, to account for variability in bull distribution across sets. All models performed better than random classification. The best model was LASSO according to classification accuracy (0.62) and Random Forest according to Area Under Curve (0.69) computed with the independent test set.

**Conclusions:**

Even with this moderate performance, our models would lead to a 12.4% point increase of the probability of identifying subfertile individuals. These results suggest that sperm DNA methylation holds potential as a relevant source of predictive markers for bull fertility.

**Supplementary Information:**

The online version contains supplementary material available at https://doi.org/10.1186/s12711-026-01097-y.

## Background

Artificial insemination (AI) with frozen semen is commonly used for dairy cow reproduction. To ensure sustainability of the sector, it is important to maintain a high rate of AI success. Bulls with lower fertility can lead to more pregnancy failures, increasing culling rates, reducing genetic gain and generating important economic losses for farmers and breeding companies. In order to increase AI success, it has been proposed to identify relevant biomarkers to select bulls with high fertility rates. In this context, statistical models have been developed to relate Single Nucleotide Polymorphisms (SNPs) to fertility in Holstein [1, 2], Jersey [3], and Brown Swiss [4] breeds. These studies rely on linear Gaussian models fitted to thousands of individuals, with correlations between observations and predictions ranging from 0.28 to 0.40. Although scientifically relevant, these models only capture the genetic component of bull fertility and their predictive ability remains moderate.

Other biological markers have been investigated to predict bull fertility. For example, spermatozoa morphological characteristics were used to predict fertility, with adjusted R² values of 0.58 and 0.73 between predicted and observed performances [5, 6]. These results, although very promising, suffer from methodological limitations. Indeed, these studies are based on a very limited number of individuals (*n* = 12 for [5] and *n* = 9 for [6]), and therefore do not allow for the implementation of a rigorous validation procedure. Similarly, an attempt to predict fertility from sperm nuclear shape achieved classification accuracy values of 91.7% for discriminating individuals categorized as Low, Medium, or High fertility [7]. However, in this study as well, the number of individuals was small (*n* = 17). Because of their small sample sizes, the results obtained in these studies are difficult to generalize to large bull populations.

DNA methylation has been reported as essential for fertility in several studies. Notably, male *Dnmt3l* knockout mice are viable but sterile, exhibiting meiotic arrest [8, 9]. Conditional *Dnmt3a* knockout in the male germline results in sterility and meiotic arrest [10], as well as a failure of spermatogonial stem cells to commit to spermatogenesis due to persistent activation of stem cell-associated genes [11]. Exposure to a detrimental environment during the *in utero* differentiation of male germ cells can lead to impaired fertility during adulthood and altered DNA methylation patterns in mature sperm [12]. Involvement of DNA methylation in spermatogenesis and its impact on male fertility and sterility, as well as the abnormal DNA methylation patterns observed in cases of male infertility, have been well documented [13].

In humans, studies have also investigated the predictive capacity of the sperm methylome in relation to male fertility [14]. Based on the methylation profiles of 127 infertile individuals undergoing in vitro fertilization treatment and 54 fertile normozoospermic men, the authors reported a 82% sensitivity (ability to identify infertile individuals). These promising results were obtained on a larger sample size than what is typically available in bulls, using a 10-fold cross-validation procedure. However, the study did not include an independent test set, and the performance reported in this work may thus be overestimated.

Interest in bull sperm DNA methylation has increased recently [15–19]; these studies all identified regions or sites in the genome showing statistical difference in DNA methylation between small groups of contrasted fertility levels. Some of the sites of interest are included in genes related to spermatogenesis and embryonic development. Although these studies suggest an association between certain DNA methylation patterns and bull fertility, they also suffer from several limitations. They are based on small sample sizes (number of individuals between 9 and 28 at most), which makes their conclusions difficult to generalize to larger cohorts. Furthermore, they did not aim to develop forecasting models capable of predicting bull fertility from DNA methylation, even though such models would be very useful for improving the reliability and success of inseminations.

In bulls, differentially methylated regions in the sperm from highly fertile bulls (sire conception rate above 50%) and low fertility bulls (sire conception rate below 40%) were used to predict sire conception rate, leading to an R² of 0.71 [19]. Although these results were obtained from a cohort of only 28 bulls, they suggest that fertility-associated markers in bulls could be used for predictive purposes. Costes et al. used a cohort of 100 Montbéliarde bulls categorized as Fertile or Subfertile and achieved a classification accuracy of 72% in predicting fertility status using a Random Forest approach; and these results could be replicated using an independent cohort of 20 bulls [20]. Although promising, all these studies are based on small datasets and rely on a limited number of predictive algorithms.

As a follow-up to this previous work, we aimed to refine this approach by testing alternative methods and applying them to AI bulls of the Holstein breed, which is globally widespread and the most commonly used breed in the dairy industry. In our study, we aim to address, as much as possible, the various methodological limitations and biases identified in the previously mentioned works, notably through a rigorous evaluation approach based on nested cross-validation. Leveraging a large dataset (*n* = 351 Holstein bulls), we compare a wide range of models (Penalized linear model regression, tree-based machine learning and neural networks) for predicting bull fertility status based on DNA methylation. To our knowledge, this is the largest cohort assembled for bull fertility prediction based on non-genetic biological markers. After identifying the best-performing models, we quantified their benefits to improve AI reliability, which is particularly important given the key role of the Holstein breed in the dairy sector.

## Methods

### Cohort, fertility index and fertility classes

This work is based on 351 commercial Holstein bulls represented by 664 frozen semen samples used for AI (for some bulls, several sperm samples were collected at different periods). For each bull, a fertility index, the non-return rate in cycle of the inseminated cows at 56 days post-insemination (NRR 56), is computed from all the AI records obtained with this bull (Additional file 1 Table S1). NRR 56 was corrected for the fixed effects of herd-year, month-year, parity of the cow, interval between calving and insemination, week day, AI technician, semen type (sexed vs. conventional), and for the genetic and permanent environmental effects of the cow. Therefore, it fully characterises the bulls and is hardly affected by other sources of variation, reflecting AI success rate at a relatively early stage of gestation [21].

**Fig. 1:**
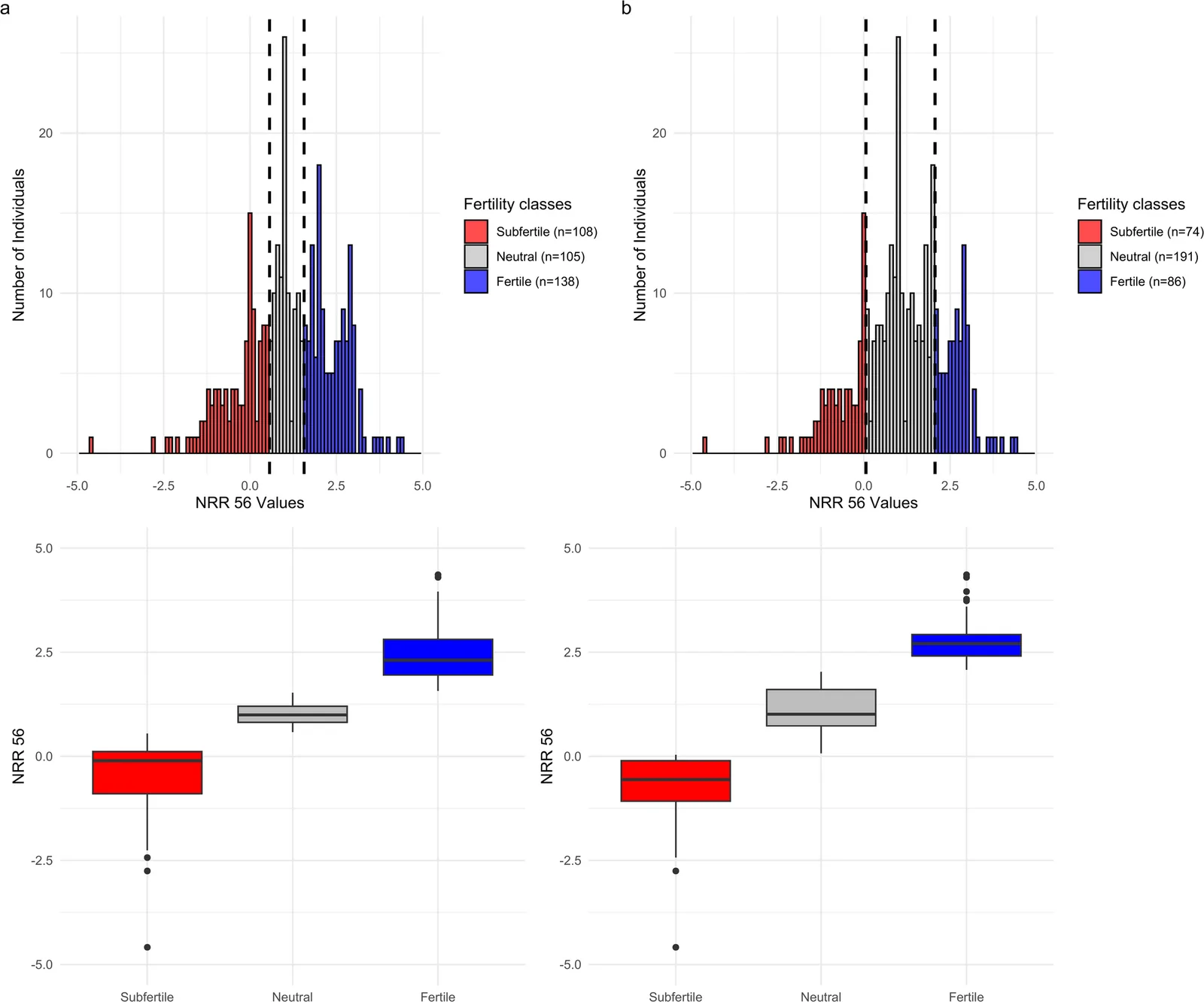
NRR 56 values and fertility classes within the cohort of bulls. Values of fertility index NRR 56 are displayed as histograms (upper panel) and boxplots (lower panel). According to their NRR 56 values, bulls were classified in three fertility categories (Subfertile, Neutral, Fertile). Only Subfertile and Fertile bulls (red and blue) are predicted by the models. (**a**) − 1-point NRR 56 difference threshold between Subfertile and Fertile groups. (**b**) − 2-point NRR 56 difference threshold between Subfertile and Fertile groups

Each bull was assigned a fertility class based on its NRR 56 value. Two types of fertility classes were defined. First, we classified bulls with NRR 56 values lower than the group median by at least 0.5 point as Subfertile (*n* = 108 bulls represented by 195 samples), bulls with NRR 56 value above the median by at least 0.5 point as Fertile (*n* = 138 bulls represented by 277 samples), and those between these two values as Neutral (Fig. 1a). Second, we defined the same three classes but with thresholds respectively below and above the median by at least 1 point, generating a 2-point difference in NRR 56 values between Subfertile (*n* = 74 bulls represented by 137 samples) and Fertile bulls (*n* = 86 bulls represented by 169 samples; Fig. 1b). The contrast between Subfertile and Fertile bulls is thus stronger in the second classification than in the first one.

The age of each bull at semen collection is reported in Additional file 1 Table S1. The cohort is predominantly composed of young bulls (median age at sampling: 19 months), which is consistent with current Holstein breeding practices, where genomic selection has substantially shortened the careers of bulls.

All bulls were sourced from two breeding companies in France. To verify that the two companies did not introduce confounding effects, we examined the distributions of bull age and NRR 56 values across companies (Additional file 2 Figs. S1 and S2). Non-parametric tests confirmed the absence of significant differences (p-value > 0.01) in age and NRR 56 distributions between companies, indicating that bull origin is unlikely to act as a confounding factor in our analyses.

### DNA methylation values and predictors

The RRBS DNA methylation data of the semen samples is described in [22]. Several filters were then applied to these data. First, we focused on a very specific subset of CpGs also targeted by a DNA methylation array specific for cattle (“RUMIGEN EpiChip” [23]), in order to lay the foundation for field applications. The intersection between RRBS data and EpiChip markers represents 21,182 CpGs. After technical filtering, excluding CpG sites with more than 20% missing values and those that are not variable across all samples, 14,285 CpGs were retained as predictive variables for the models (Additional file 1 Table S2). Considering the two different fertility classes defined above, we defined two CpG datasets, one CpG dataset for the 1-point fertility contrast (246 bulls) and another CpG dataset for the 2-point fertility contrast (160 bulls). Each CpG dataset contains 14,285 columns corresponding to the DNA methylation values (in the range 0–1) of the 14,285 CpGs, and either 472 (dataset 1) or 306 (dataset 2) rows corresponding to the number of semen samples from 246 (dataset 1) and 160 (dataset 2) bulls (Additional file 1 Tables S3 and S4).

The large number of potential predictors (methylation levels of the CpGs) compared to the number of individuals makes the development of predictive models very challenging. Principal Component Analysis (PCA) was implemented to reduce the number of predictors. Importantly, the PCA was performed from the methylation data matrix (DNA methylation values for all samples and CpG loci), without any use of the fertility phenotype. Its purpose was solely to reduce the number of predictors while preserving the variance structure, by transforming the original CpG methylation variables (14,285) into independent principal components (PCs), i.e., uncorrelated linear combinations of the initial CpG variables. Mathematically, the number of PCs cannot exceed the number of samples and, for this reason, the number of PCs was thus set equal to 471 and 305, depending on the dataset considered. These 471 and 305 PCs were used as new predictors to build the predictive models (Additional file 1 Tables S5 and S6).

### Classification models

Four standard supervised learning algorithms were employed: Least Absolute Shrinkage and Selection Operator (LASSO), Random Forests (RFs), Gradient Boosting (GB) and Neural Network (NN). Short descriptions of these models and their main parameters are presented below.

#### LASSO

LASSO is a machine learning algorithm, relying on a penalized linear or generalized regression method [24]. With LASSO, a penalty L1 is applied to prevent overfitting. Thanks to the penalty, the algorithm is able to assign a 0-coefficient to predictors (here, CpGs or PCs) that have low impact on the classification. This way, the number of variables actually included in the model may decrease drastically when only few predictors appear to be relevant. In terms of interpretation, if the regression coefficient associated to a predictive variable is positive, it is associated with a higher probability of getting a fertile individual. If the regression coefficient is negative, it is associated with a reduced probability of getting a fertile individual. If the value is zero, then the predictor has no impact and is therefore excluded from the model. LASSO models were trained using “glmnet” [25–26] and “caret” [27] packages in R. The hyperparameter λ, governing the penalty level with which the L1 penalty is applied on regression coefficients, was optimized through a cross-validation procedure within the training data detailed below. Values were selected to optimize classification accuracy in the testing fold among 1000 λ values at regular intervals between 0.001 and 0.2.

#### Random forests

RFs are based on ensembles of Classification And Regression Trees (CART). CART is classifying individuals in the two groups (Subfertile vs. Fertile) using successive splitting rules based on the predictors (CpGs or PCs). The best splitting rules are selected to minimize classification errors during training. Different CARTs are generated by sampling different subsets of individuals and predictors from the training set (bagging process). RF builds thousands of such trees and then aggregate their outputs for classifying individuals. RF models were trained using “randomForest” [28] and “caret” [27] packages in R. Two hyperparameters were optimized during the cross-validation within the training data: (i) the number of trees built to generate the predictive model (ntree) and (ii) the number of predictors randomly selected to make a splitting rule for each node formation (mtry). The value of *ntree* was set to 5000 for all four datasets tested, while the *mtry* parameter was adapted to each dataset. For each case, tested *mtry* values ranged from 1 to the maximum number of predictors in the dataset (305 and 471 for PC, and 14,285 for CpG), with five values evaluated at regular intervals across this range.

#### Gradient boosting

GB is also based on regression trees, but each new tree is built using information from the previously generated one (while RF trees are designed to be as independent from each other as possible). Initially, a single tree with limited predictive power is constructed; subsequent trees are then built sequentially to correct the errors of the preceding ones. GB models were trained using “xgboost” [29] and “caret” [27] packages in R. Three hyperparameters were optimized: the number of trees to build the model (nrounds), depth of the trees (max-depth), and the learning rate of the model (eta). To limit computational demand, the set of parameter combinations tested was reduced. The values of nrounds considered were 200, 300, 400, and 500; max_depth values ranged from 4 to 6; and the tested eta values were 0.001 and 0.01.

#### Neural networks

NNs consist of layers of interconnected nodes neurons, where each layer transforms the outputs of the previous layer using weights and an activation function. We use the standard “relu” activation function for the hidden layers and the “softmax” activation function for the final layer in order to classify the individuals into either the Fertile or Subfertile class. The softmax function transforms the raw output scores (logits) of the final layer into a probability distribution over classes. The NNs models were trained using the “Keras” [30] package in R. Three hyperparameters were optimized: the number of hidden layers in the model, the number of neurons in each layer, and the learning rate of the model. The learning rates evaluated were 0.0001, 0.001, and 0.01. The number of hidden layers tested was 2 or 3, with each layer allowed to contain 16, 32, or 64 neurons.

### Model evaluation

By combining the four different datasets and the four machine learning algorithms, we defined 16 models. For each dataset considered, 80% of individual bulls were used in the training set, and the remaining 20% were used in the test set. Twenty different training and test sets (same for the four algorithms) were randomly generated according to the above procedure. The group separation was randomly done on bulls. Only one sample from each bull was included in the test set, to prevent artificial inflation of the predictive quality (with one bull being correctly predicted and represented several times) or overfitting during the learning process. For bulls represented by several samples and assigned to the test set, one sample was randomly selected at each iteration; all samples were retained for bulls assigned to the training set.

**Fig. 2:**
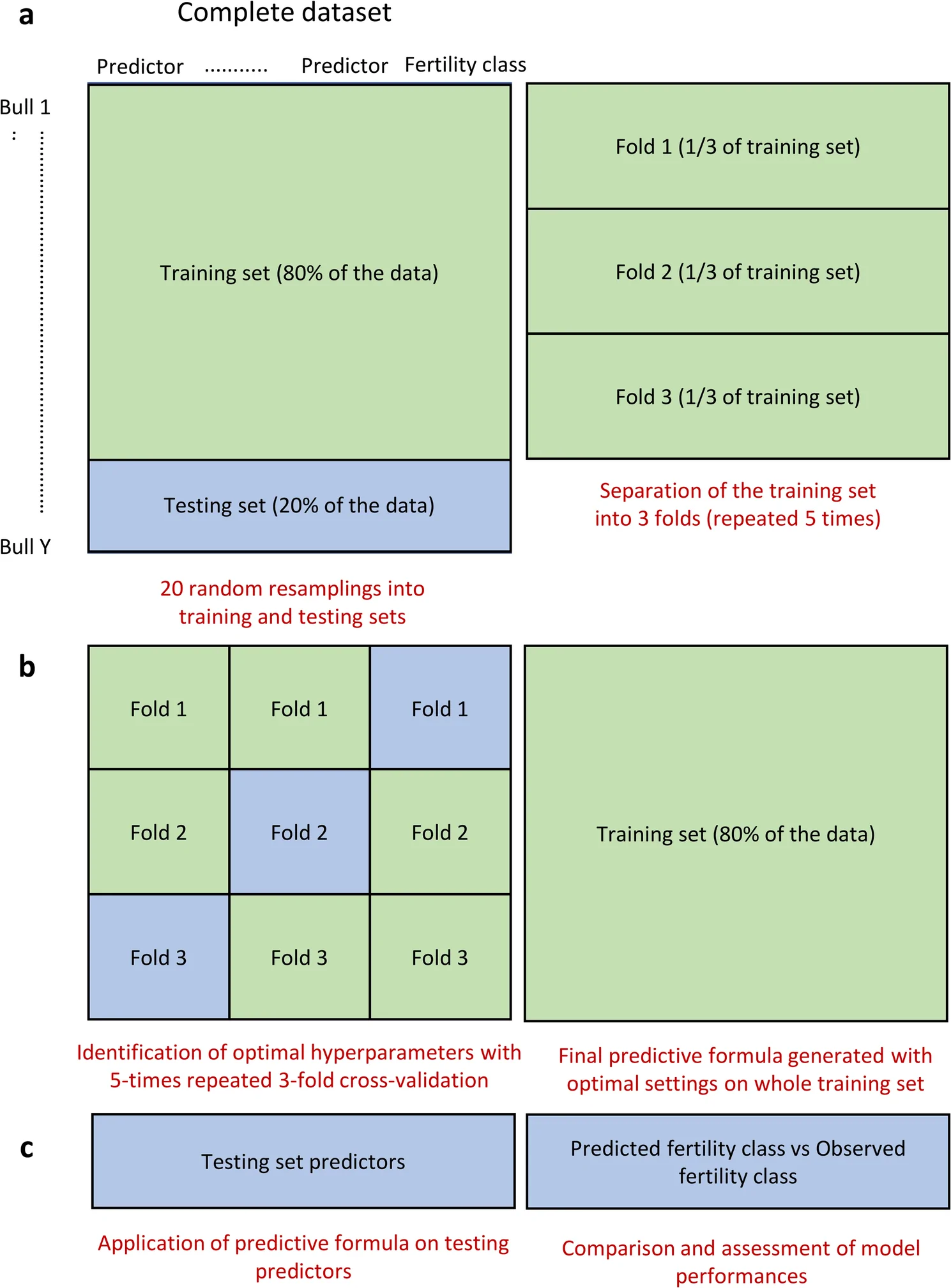
Global workflow for the modelling process. (**a**) - The full dataset is initially split into training and test sets. The training and test sets are respectively constituted of 80% and 20% of the full dataset. This initial splitting is repeated 20 times. To prepare cross-validation, training set is then split into 3 folds (process iterated 5 times). (**b**) - The cross-validation procedure is used to determine optimal hyperparameters values for each algorithm. Each fold is used alternatively to determine optimal hyperparameters (process repeated for the 5 combinations of fold). The predictive formula is then generated with optimal settings on the whole training set. (**c**) - The tuned algorithm is then applied on predictors of the test set in order to generated predicted fertility class. Predicted fertility class and observed fertility class are then compared to evaluate the model performances

In order to optimize the values of hyperparameters, we performed a repeated (five times) three-fold cross validation (CV), to limit as much as possible the risk of overfitting. Figure 2 illustrates the whole CV process. The training sample set was randomly divided into three subgroups called folds. A predictive model was built using a specific set of hyperparameter values on two folds and its performance was evaluated on the remaining one. For each combination of hyperparameters, this process was repeated twice, each time using a different fold to evaluate performances. CV was repeated five times, using different splittings. The performance values obtained for each set of hyperparameters were then averaged over the five repetitions. The hyperparameters combination leading to the highest classification accuracy (see below) for each algorithm was then retained. The corresponding mean classification accuracy over the cross-validation folds is hereafter referred to as the training classification accuracy. It is reported to assess overfitting, by comparison with the classification accuracy obtained on the independent test set. As it is computed on the folds used for hyperparameter selection, this value is expected to be slightly optimistic. Each algorithm was then trained again using the whole training dataset (including 80% of the bulls) with the selected hyperparameters values. The trained algorithms were then used to predict the fertility classes of the bulls of the test dataset (20% of the individuals; Fig. 2).

The bulls of the test dataset were then allocated into four categories: Subfertile bull predicted as Subfertile (true positive), Subfertile bull predicted as Fertile (false negative), Fertile bull predicted as Subfertile (false positive), and Fertile bull predicted as Fertile (true negative). Several standard performance indicators, namely, classification accuracy, sensitivity, specificity and Area Under Curve (AUC) were computed from the results. Classification accuracy corresponds to the number of correctly predicted bulls divided by the total number of bulls. It reflects the global performance of the algorithm without focusing on a specific class. Sensitivity corresponds to the number of Subfertile bulls correctly predicted divided by the total number of Subfertile bulls, reflecting the capacity of the model to correctly identify Subfertile bulls. Specificity corresponds to the number of Fertile bulls correctly predicted divided by the total number of Fertile bulls, reflecting the capacity of the model to correctly identify Fertile bulls. To compute classification accuracy, sensitivity and specificity, each bull was assigned to the Subfertile class when its predicted probability of being Subfertile was ≥ 0.5, and to the Fertile class otherwise. AUC measures the model’s overall ability to distinguish between classes. For each bull of the test set, the predictive model provided a predicted probability of being Subfertile. The ROC curve was obtained by plotting sensitivity against 1 − specificity for all possible probability thresholds used to assign bulls to the Subfertile class, and the AUC was computed as the area under this curve. The AUC corresponds to the probability that a randomly chosen Subfertile bull receives a higher predicted probability than a randomly chosen Fertile bull; it is therefore independent of the classification threshold. An AUC of 0.5 corresponds to random classification and an AUC of 1 to perfect discrimination. AUC values were then averaged across the 20 iterations.

A Kruskal–Wallis test was used to identify the model characteristics (type of markers, fertility gap, algorithm, initial partitioning into training and test set) with significant effects on AUC. If a significant effect (p-value < 0.01) was detected for a given model characteristic, pairwise comparisons were conducted using Dunn’s test with a Bonferroni correction to account for multiple testing. These analyses were carried out in R using the « rstatix » package [31].

The Positive Predictive Value (PPV) metric represents the probability that a bull is actually Subfertile when our model predicts it as Subfertile. PPV is defined as follows:

1 $${\rm{PPV = }}{\matrix{ {\rm{sensitivity*Subfertile}} \hfill \cr {\rm{bulls prevalence}} \hfill \cr} \over \matrix{ \left( {{\rm{sensitivity*prevalence}}} \right) \hfill \cr {\rm{ + }}\left( {{\rm{1 - specificity}}} \right){\rm{*}}\left( {{\rm{1 - prevalence}}} \right) \hfill \cr} }$$

## Results

The objective of this study is to compare the ability of various machine learning models to discriminate between subfertile and fertile bulls. To this end, we employed 4 different algorithms, run on four datasets (two types of predictors and two fertility gaps to define the fertility classes), resulting in a total of 16 different models tested. Given the still relatively limited size of our cohort, we repeated the model performance evaluation procedure 20 times, using 20 different combinations of bulls in the training and test sets, to assess the impact of random partitioning on model performance.

### Assessment of genetics and age as confounders

Because model performance could be influenced by the genetic structure of our population, pairwise genetic relatedness was calculated among all bulls in the cohort. The genomic relationship coefficients did not reveal any systematic enrichment or population stratification within the fertility groups, as within-group relationship values were very similar to between-group values (Additional file 2 Fig. S3). This observation rules out any major bias related to population structure or family-based prediction.

Because age has a major effect on sperm DNA methylation [32], we first evaluated the potential confounding effect of age across the training and test sets to ensure the validity of our predictive models. Wilcoxon tests and quantile–quantile analyses showed no significant differences in age distributions between groups (all p-values > 0.01) for both the 1-point and 2-point NRR 56 gap definitions. These results confirm the absence of systematic age imbalance across the 20 resampling iterations (Additional file 2 Fig. S4).

These results, together with the absence of significant differences in NRR 56 and age between the two breeding companies of origin (Additional file 2, Figs. S1 and S2), suggest that major confounding factors are unlikely to substantially influence model performance. We therefore did not attempt to incorporate multiple, potentially interrelated effects into the models, given the limited environmental information available, and instead focused on assessing the predictive value of DNA methylation per se.

### Model performances

Table 1 summarizes the performances of each model averaged over the 20 iterations. Among the 16 models tested, substantial disparities are observed across all indicators, with several emerging trends.

**Table 1:** Performance indicators for all tested models

Type of markers	Fertility gap	Algorithm	Train classification accuracy	Test classification accuracy	Test sensitivity	Test specificity	Test AUC
CpG	2 points	LASSO	0.765 [0.750;0.780]	0.622 [0.582;0.662]	0.682 [0.620;0.743]	0.586 [0.508;0.665]	0.669 [0.627;0.710]
CpG	2 points	RF	0.745 [0.735;0.756]	0.617 [0.587;0.647]	0.737 [0.683;0.791]	0.499 [0.425;0.572]	0.653 [0.620;0.686]
PC	2 points	RF	0.731 [0.720;0.742]	0.609 [0.568;0.650]	0.719 [0.656;0.782]	0.498 [0.424;0.572]	0.693 [0.652;0.735]
PC	1 point	RF	0.695 [0.688;0.702]	0.585 [0.559;0.610]	0.830 [0.787;0.872]	0.284 [0.227;0.340]	0.605 [0.574;0.637]
PC	2 points	GB	0.711 [0.701;0.721]	0.584 [0.557;0.612]	0.709 [0.663;0.754]	0.446 [0.388;0.504]	0.619 [0.587;0.650]
CpG	1 point	RF	0.723 [0.716;0.730]	0.584 [0.560;0.607]	0.792 [0.754;0.830]	0.327 [0.288;0.367]	0.590 [0.563;0.617]
CpG	2 points	GB	0.726 [0.714;0.738]	0.581 [0.545;0.618]	0.654 [0.586;0.723]	0.533 [0.444;0.621]	0.614 [0.581;0.647]
PC	2 points	LASSO	0.703 [0.690;0.716]	0.577 [0.543;0.610]	0.720 [0.678;0.761]	0.428 [0.366;0.490]	0.589 [0.553;0.624]
PC	2 points	NN	0.639 [0.629;0.650]	0.572 [0.529;0.615]	0.641 [0.576;0.706]	0.507 [0.412;0.602]	0.591 [0.557;0.625]
CpG	1 point	NN	0.677 [0.671;0.684]	0.568 [0.542;0.595]	0.669 [0.537;0.801]	0.433 [0.282;0.584]	0.590 [0.556;0.624]
CpG	1 point	LASSO	0.735 [0.727;0.743]	0.568 [0.545;0.592]	0.712 [0.670;0.754]	0.393 [0.353;0.433]	0.562 [0.530;0.594]
CpG	2 points	NN	0.681 [0.668;0.694]	0.564 [0.513;0.615]	0.730 [0.574;0.886]	0.427 [0.264;0.589]	0.588 [0.535;0.641]
PC	1 point	LASSO	0.669 [0.661;0.677]	0.563 [0.535;0.591]	0.772 [0.721;0.824]	0.301 [0.248;0.354]	0.521 [0.496;0.546]
CpG	1 point	GB	0.726 [0.718;0.734]	0.558 [0.535;0.581]	0.730 [0.693;0.767]	0.343 [0.303;0.383]	0.566 [0.541;0.591]
PC	1 point	NN	0.654 [0.649;0.659]	0.547 [0.523;0.571]	0.645 [0.600;0.689]	0.423 [0.371;0.475]	0.569 [0.545;0.593]
PC	1 point	GB	0.693 [0.685;0.701]	0.535 [0.506;0.563]	0.718 [0.683;0.752]	0.308 [0.263;0.353]	0.548 [0.522;0.573]

The results are ranked in descending order of test classification accuracy, and in case of a tie, by descending order of AUC. PCs correspond to the principal components value extracted as described in the Material and Methods section. Values in brackets indicate the 95% confidence interval for each performance indicator. Classification accuracy measures the proportion of correctly classified bulls and is defined as: Classification accuracy=$\:\frac{TP+TN}{TP+TN+FP+FN}\:$, where TP, TN, FP, and FN correspond to true positives, true negatives, false positives, and false negatives, respectively. Sensitivity quantifies the ability of the model to correctly identify Subfertile bulls and is calculated as: Sensitivity=$\:\frac{TP}{TP+FN}$. Specificity reflects the ability to correctly identify Fertile bulls and is defined as Specificity=$\:\frac{TN}{TN+FP}$. Finally, the AUC summarizes model performance across all classification thresholds, with values ranging from 0.5 (random classification) to 1 (perfect classification)

The AUC values range from 0.548, observed for the GB algorithm applied to the PCs and 1-point fertility gap dataset, to 0.693, obtained with RF on the PCs and 2-point fertility gap dataset. This 0.145 point gap between the best- and worst-performing models indicates substantial differences in predictive power. Between these two extremes, model performances are relatively continuously distributed. Although limited, the performances of all models are above what would be expected by chance (AUC = 0.5), suggesting that the methylome has the potential to predict fertility of Holstein bulls. Model classification accuracy ranges from 0.535 to 0.622, with lower values and a smaller spread than AUC values. Unlike AUC, which integrates performance over all possible probability thresholds, classification accuracy, sensitivity and specificity depend on the threshold used to assign bulls to a class (0.5 in this study). In addition, a striking imbalance is observed between sensitivity, which ranges between 0.641 and 0.830, and specificity, which is below 0.586. This result strongly suggests that whatever the model, subfertile bulls are more easily predicted than fertile bulls. Another key point to mention is the substantial gap between training and test performances. This gap ranges from 0.067 to 0.167. Importantly, the confidence interval is wider for the test classification accuracy than for the training classification accuracy, indicating strong variations in the ability of the trained models to accurately predict fertility of an independent bull population.

### Algorithms, fertility gap and splittings influence model performances

**Fig. 3:**
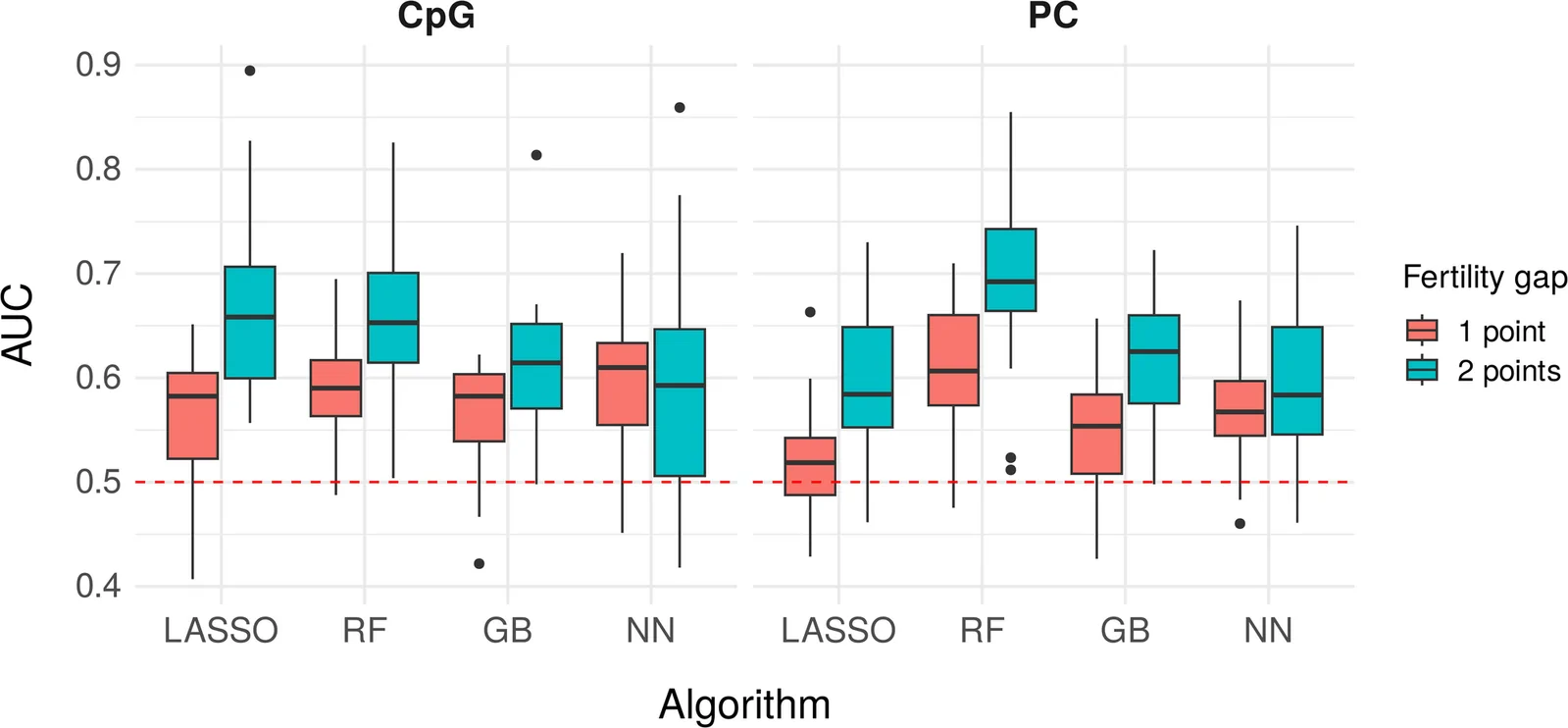
AUC of the predictive models. Each boxplot represents the test AUC values obtained across the 20 partitionings for each of the 16 models

We next investigated the factors that significantly impact the model performances. To this end, we assessed the effect of the nature of the predictors (CpG vs. PC), the algorithm (RF, LASSO, GB, NN) and the fertility gap (1-point vs. 2-point) on the test AUC value using a non-parametric test. Because of the important variation of the test classification accuracy (Table 1) and the test AUC (Table 1; Fig. 3; reflected by the sizes of the boxplots), we also investigated the effect of the initial partitioning between the training and test sets on the performances. This effect was captured through the twenty distinct random training/test partitioning. When performing the non-parametric test, the AUCs obtained with different data partitions were significantly different (p-value = 0.0068, Table 2), indicating that this initial split has a significant impact on the models’ performance. However, when doing pairwise comparisons between random data partitioning, only two display significant differences. This result suggests that the effect of the data partitioning remains overall moderate, although important overestimation or underestimation of the model performances due to the initial partitioning can be observed in rare cases, probably due to the small population size.

**Table 2:** Factors influencing performances

Features	Df	*P*-value
Data partitioning	19	0.0068
Fertility gap Type of predictors Algorithms	1 1 3	7.48 × 10^–10^ 0.117 2.3 × 10^–10^

The effect of four factors on the test AUC was assessed using a non-parametric test (data partitioning, predictor type, fertility gap and algorithm). The results of this test are indicated in the table

Among other factors, it appears that the predictor type does not have a significant impact on the performance achieved (p-value = 0.117). This observation is particularly interesting, given that it is generally acknowledged that a high number of predictive variables relative to the number of samples tends to limit the performance of machine learning approaches. In contrast, the choice of algorithm has a strong impact (p-value = 2.3 × 10⁻⁵). Since we used four different algorithms, we performed pairwise comparisons between all algorithm pairs and found that RF outperforms the other three algorithms (LASSO, GB and NN). It should be noted that for pairwise comparisons between LASSO, GB, and NN, no significant performance differences were observed (p-value > 0.68 at least). The fertility gap showed a highly significant effect (p-value = 7.48 × 10⁻¹⁰), reflecting better performance for models with a larger fertility gap between the two groups, compared to those that include more individuals but with smaller gaps. Ultimately, of the four characteristics assessed, three, random partitioning, algorithms, and fertility gap, have a significant influence on model performance, while the nature of the input predictors appears to have a comparatively minor effect.

We next conducted non-parametric tests on all model parameters regarding gap in training and testing classification accuracy to determine whether certain model characteristics made them more susceptible to the performance drop during model evaluation. Among all the factors tested, only the predictor type significantly affected the performance loss between training and testing (p-value = 0.009), indicating that PCs are less prone to this drop than CpGs (with an average classification accuracy loss of 0.0975 for PCs versus 0.13 for CpGs).

### Relationship between model performance and training set size

To assess the dependence of model performance on the training set size, we conducted a learning curve analysis by progressively reducing the training dataset from 100% down to 20% of its original size, while keeping the validation test set unchanged. This analysis was performed using the top-performing operational configuration (CpG, 2-point fertility gap, LASSO classifier). As shown in Additional file 2 Fig. S5, the mean AUC increases steadily from the smallest training set size (20% of full size, mean AUC = 0.55) through intermediate steps (40–80% of full size, mean AUC ranging from 0.62 to 0.65), reaching its peak with the full training dataset (AUC = 0.67). Notably, the rate of improvement slows down significantly when the training set size exceeds 60%, indicating that while further expansions of the cohort could potentially enhance robustness, the marginal gains in classification accuracy may be limited.

### CpG markers heritability

Because sperm DNA methylation is partly controlled by genetic factors [22], we evaluated whether the predictive performance of the top-performing model primarily relies on underlying genetic factors influencing the methylome. To this end, we examined the heritability of the CpG sites selected by the model (CpG, 2-point fertility gap, LASSO) across the 20 resampling iterations. Selected CpGs were categorized according to their frequency of selection in the models: Singletons (selected once), Low (2–5 times), Medium (6–10 times), and High (11–20 times). The median heritability of these groups ranged from 0.19 to 0.26 (0.19 for singletons, 0.23 for the low category, and 0.26 for both medium and high categories), which is consistent with or slightly above the overall median value observed across all CpGs considered in the study (median = 0.19) (Additional file 2 Fig. S6). While a few highly recurrent markers exhibited slightly higher heritability values, the absence of a sharp contrast or over-representation of highly heritable CpGs demonstrates that the predictive performance of the model is not explained exclusively by the genetic component of DNA methylation.

### Potential benefits for the artificial insemination sector

Regarding the needs of the agricultural sector, the most relevant selection criterion is a model’s ability to correctly identify subfertile bulls, a metric captured by sensitivity in our study. The best-performing model in terms of sensitivity reached a 0.830 value (RF, PC, 1-point); however, it exhibited very low specificity (0.285), indicating a tendency to classify nearly all bulls as subfertile. Among all tested models, three stand out by combining a decent sensitivity, around 0.700, a classification accuracy above 0.600, and an AUC above 0.650, reflecting a reliable ability to detect subfertile bulls without systematically misclassifying all individuals. These are the most balanced models identified in our study, offering higher classification accuracy and specificity than others, and thus a better trade-off between all performance metrics. These three models are: (i) LASSO, CpG, 2-point (sensitivity = 0.682, specificity = 0.586); (ii) RF, CpG, 2-point (sensitivity = 0.737, specificity = 0.499); and (iii) RF, PC, 2-point (sensitivity = 0.719, specificity = 0.498). Since model (ii) outperformed model (iii) for both indicators, we excluded it for the following analysis. To evaluate potential benefits for the breeding sector, we computed the PPV of these two models as a function of the prevalence of subfertile bulls in the cohort. Indeed, since our model is not based on identifying the actual NRR 56 values directly, but only on fertility classes, we propose to assess the benefit of the model in terms of its ability to eliminate individuals with the lowest fertility (Subfertile), in the context of a potential field selection. We performed this evaluation for our two models and measured the benefit each approach would provide compared to a random model.

**Fig. 4:**
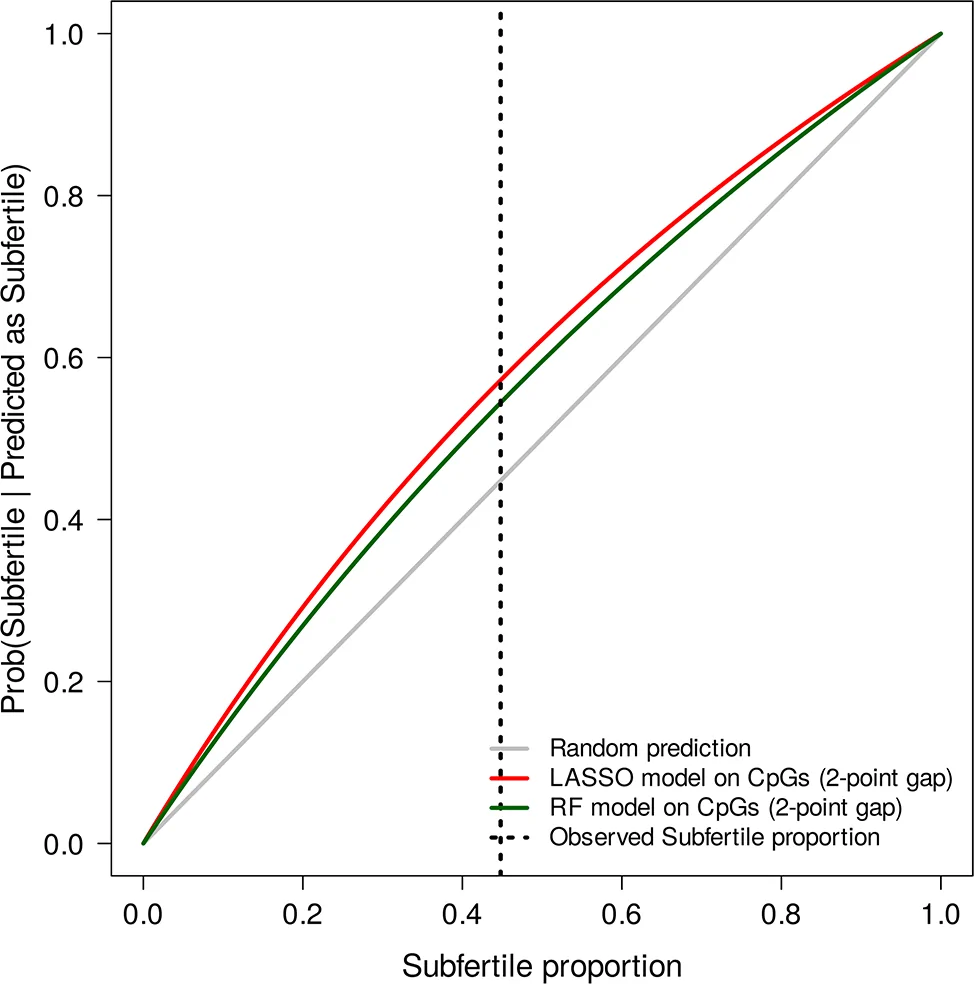
Positive predictive value (PPV) as a function of subfertile bulls’ proportion. For both LASSO and RF models based on CpGs with a two-point fertility gap, the ability to identify subfertile individuals is higher than random classification. In our study, the prevalence of subfertility is 0.448 and, for this prevalence, the PPVs are 0.448, 0.572, 0.544; for the random model, the LASSO model based on CpGs with a fertility gap of 2 points, and the RF model based on CpGs with a fertility gap of 2 points, respectively

In our study, the proportion of subfertile bulls is 0.448, which corresponds to an interesting value range for applying the strategy of eliminating subfertile bulls, compared to more extreme distributions where the gain would be much smaller. By definition, the PPV of the random model corresponds to the prevalence of Subfertile bulls in our study (0.448). This value serves as the reference against which we compare our two best-performing models. The LASSO model (i) has a PPV of 0.572 and the RF model (ii) has a PPV of 0.544. This means that for these two models, we would obtain respective gains of 12.4 and 9.6% points in the ability to identify subfertile individuals. In the context of selecting bulls based on their fertility, this represents a significant contribution to the ability to eliminate the least performing bulls. Figure 4 illustrates the gain in subfertile bull identification thanks to models (i) and (ii).

## Discussion

We observed that the best models show promising performances, with maximum AUC of 0.693 and classification accuracy of 0.622. These results are relatively consistent with those obtained using a similar approach in the Montbéliarde cattle by Costes et al. (2022) [20], who reported a classification accuracy of 0.72. The lower classification accuracy observed in the present study may be explained by methodological differences. In particular, Costes et al. selected the predictive variables on the same dataset as the one used for prediction, which can inflate performance; the larger size of our cohort allowed us to avoid this approach. Furthermore, their modelling approach involved only a single test set, in contrast to the 20 different partitions considered in our study. Given the range of performances observed across our iterations, their results can be considered similar compared to those obtained here. In contrast to studies based on sperm morphology or nuclear shape [5–7], which reported high R² or classification accuracy values (> 0.58 or > 0.90) but relied on very small sample sizes (*n* ≤ 17), our results are based on a substantially larger cohort, making them more robust and generalizable. Also, our primary objective was to identify potentially subfertile bulls in an operational framework relevant to breeding companies.

Although the models’ performances remain moderate, our results remain promising in the context of bovine prediction, especially considering that fertility variation in commercial bulls used for insemination is much narrower than in other species such as humans, since individuals with severely impaired fertility are typically removed through the selection process. This could explain the better performance of models based on a larger fertility gap of 2 points compared to those using a 1-point difference, even though the former approach involves fewer animals. Also in our study, the maximum AUC reached 0.693, whereas the maximum classification accuracy was only 0.622. The latter corresponds to the performance achievable under practical application conditions. Since classification accuracy was computed with a fixed probability threshold of 0.5, whereas AUC integrates all possible thresholds, optimizing this threshold could lead to a more effective model in practice, thereby reinforcing the relevance of this predictive approach based on bull methylome data.

Furthermore, in our study, we rely on DNA methylation markers present on the RUMIGEN EpiChip, a DNA methylation array developed as part of the European RUMIGEN project, which could serve as a tool for the routine measurement of DNA methylation in cattle. Promising results could be achieved using only one third of the markers included in the EpiChip. This reinforces the practical value of our approach. Moreover, the large-scale use of this tool, including for the prediction of other phenotypes, could help increase the size of the bull cohort with methylation data, thereby allowing to refine our models and achieving better performances.

By selecting the three most promising models, based on the evaluation criteria described in the Results section, we demonstrated that our approach enables the elimination of an additional 12.4% points of subfertile individuals compared to a random selection strategy. While this may seem moderate, these bulls are used on a very large scale, with between 127 and 64,826 artificial inseminations per bull in our study. Given the high number of AIs performed, eliminating 12.4% points more subfertile bulls could represent a significant economic gain. Although our study does not directly quantify this economic benefit, our findings nonetheless pave the way for future studies aimed at real-world application.

Due to our approach, which focuses on defining fertility classes, it is much more complex to compare our results with genetic models that directly predict fertility indices and report R² values [1–4]. However, some methodological insights can be drawn from our work, particularly the need for a very rigorous evaluation procedure. Indeed, while many studies do not include an independent cohort as a test set, we observed a strong gap between the performance on the training and test sets, as well as a moderate but significant effect of the initial partitioning between the training and test sets. These results emphasize not only the need for an independent cohort, which is essential to truly assess model performance without bias, but also the need to perform iterated tests. This leads us to believe that our reported performance is likely closer to real-world expectations than results obtained from studies based on smaller sample sizes. The overfitting phenomenon we observed may also be reflected in the overall better performances of the RF algorithm compared to the others, as its underlying mechanism makes it more robust to overfitting than other algorithms. It should be noted, however, that the GB and NNs models used here were built using a relatively limited range of hyperparameters, which may have restricted the full potential of these approaches.

From a methodological perspective, it is also worth noting that the PCs do not perform worse than the CpGs. The use of PCs greatly reduces the computational resources required for modelling, an important factor to consider in a perspective of field application of these approaches to large-scale datasets. Another point to highlight is the significant discrepancy in the ability to identify subfertile bulls compared to fertile ones. This systematic trend may suggest the existence of an epigenetic signature specific to subfertile bulls. Further investigations are needed to support this hypothesis, as the biological mechanisms underlying our results were not explored in our study.

An important question raised during the evaluation of these models is whether their predictive performance primarily reflects genetically controlled variation in DNA methylation. To investigate this, we cross-referenced the CpGs selected by our top-performing operational configuration (CpG, 2-point fertility gap, LASSO) with a recent methylome-wide association study in cattle sperm [22], stratifying them by their selection frequency across the 20 resampling iterations. The median heritability of these CpG groups (ranging from 0.19 for singletons to 0.26 for medium and highly recurrent markers) closely aligned with or was only marginally higher than the baseline heritability observed across the entire CpG set (median = 0.19; Additional file 2 Fig. S6). In addition, pairwise genomic relatedness coefficients showed no systematic enrichment within fertility classes. Together, these findings demonstrate that our machine learning models are probably capturing multi-factorial epigenetic signatures rather than a confounding latent family structure or purely additive genetic effects. This is further supported by the inherently low heritability of field fertility in Holstein cattle (typically h² < 0.05), which naturally limits the standalone predictive power of genomic selection for this specific trait and highlights the complementary value of sperm epigenetic biomarkers. Although not performed in the present study, a genomic-only prediction model built on the same cohort would be a valuable next step, as a baseline against which the added value of methylation could be benchmarked. In addition, genetic markers could potentially be combined with epigenetic markers in the same model to improve future prediction performance.

Although our study attempts to address as many limitations as possible from previous work on bull fertility, several constraints remain. The first is the cohort size itself, which is still limited in fully leveraging the potential of machine learning models. Accumulating more data through the use of the EpiChip could help reduce this bias in the future, although this issue is likely to persist for some time.

Also, the definition of fertility classes, while necessary for operational selection decisions, is relative and open to discussion, as fertility levels are defined arbitrarily, in a relative manner, and without formal criteria for class assignment. We acknowledge that it would be interesting to predict the continuous NRR 56 index instead of performing binary classification. Regression-based approaches predicting NRR 56 quantitatively represent a natural extension of our work and will be considered in future studies.

The predictive models developed here are specific to Holstein bulls. So far, Holstein is the only breed in France for which it has been possible to gather a substantial number of semen samples from bulls of relatively homogeneous age. Given the global importance of Holstein dairy cattle, our breed-specific model remains of substantial practical value. However, developing models applicable to other breeds would be of great interest as well in follow-up studies.

Additionally, fertility is a multifactorial and complex phenotype, and our study is based on only a very limited portion of the bovine methylome. In this regard, it is not surprising that our model does not achieve very high performance. It should also be noted that our study does not delve into the specific methylation signatures highlighted by the models, nor into the underlying biological mechanisms. Exploring these aspects could provide valuable insights into the processes leading to the establishment of the fertility phenotype.

## Conclusions

Our study evaluated the ability of four machine learning algorithms to distinguish fertile from subfertile bulls within a cohort of 351 individuals, using partial DNA methylome data. The models achieved AUC of 0.693 and classification accuracy of 0.622, which is better than would be expected by chance. These results reinforce the predictive potential of the bovine methylome, although it remains insufficient for fully predicting the complex phenotype of fertility. From an applied perspective, the top-performing models enabled the identification of 12.4% points more subfertile bulls than a random classification. This improvement could translate into significant economic benefits in large-scale breeding programs. From a methodological perspective, this study highlights the necessity of rigorous evaluation procedures, involving sufficiently large and independent cohorts, as well as the selection of machine learning algorithms that are adapted to the biological context of the prediction task.

## Electronic Supplementary Material

Below is the link to the electronic supplementary material.

Supplementary Material 1

Supplementary Material 2

## Data Availability

RRBS fastq files are available through the NCBI SRA under accession number PRJNA1225939 (http://www.ncbi.nlm.nih.gov/bioproject/1225939). All processed data supporting the results are provided in the manuscript and supplementary materials.
