# Supplementary material for: Assessing the predictive potential of the Holstein bovine sperm methylome for bull fertility using machine learning
Source: Genet Sel Evol. 2026 Oct 30;58:77. doi: 10.1186/s12711-026-01097-y (PMC13723140; doi:10.1186/s12711-026-01097-y)
Supplement: Supplementary file 1 [file 12711_2026_1097_MOESM2_ESM.docx]

**Additional file 2 – Supplementary Figures**

**Assessing the predictive potential of the Holstein bovine sperm methylome for bull fertility using machine learning**

A. Asset*^1,2^*, V. Costes*^3^*, C. Fouéré*^3,4^*, A. Raja-Ravi-Shankar*^1,2^*, C. Le Danvic*^3^*, F. Jaffrezic*^4^*, H. Kiefer*^1,2^*^#^*, D. Makowski*^5^*^#^

*^1^*Université Paris-Saclay, UVSQ, INRAE, BREED, Jouy-en-Josas, France; ^2^Ecole Nationale Vétérinaire d’Alfort, BREED, Maisons-Alfort, France; *^3^*Eliance, Paris, France; *^4^*Université Paris-Saclay, INRAE, AgroParisTech, GABI, Jouy-en-Josas, France; *^5^*Université Paris-Saclay/INRAE/ AgroParisTech, UMR MIA Paris-Saclay, Palaiseau, France

^#^These authors equally contributed to this work

*Corresponding author

E-mail addresses:

AA: alexandre.asset@inrae.fr

VC: valentin.costes@eliance.fr

CF: corentin.fouere@eliance.fr

ARRS: amrita.raja-ravi-shankar@inrae.fr

CLD: chrystelle.ledanvic@eliance.fr

FJ: florence.jaffrezic@inrae.fr

HK: [helene.kiefer@inrae.fr](mailto:helene.kiefer@inrae.fr)

DM: david.makowski@inrae.fr

**Figure S1.** Distribution of bull NRR 56 according to breeding companies. No significant difference (p > 0.01) in NRR 56 distribution was observed between breeding companies using non-parametric tests.

**Figure S2.** Distribution of bull ages at semen collection in breeding companies. No significant difference (p > 0.01) in age distribution was observed between breeding companies using non-parametric tests.

**Figure S3.** Distribution of pairwise relatedness coefficients among bulls, stratified by fertility group membership (within-group vs. between-group). No systematic enrichment of genetic relatedness within fertility groups was observed. Fert, fertile bulls; sub_fert, subfertile bulls.

**Figure S4.** See also next page.

**Figure S4.** See also previous page. Quantile–quantile plots comparing age distributions between groups in the training and test sets for the 1-point and 2-point NRR 56 gap definitions. Wilcoxon rank-sum tests revealed no significant differences across the 20 resampling iterations (all p > 0.01).

**Figure S5.** Learning curve analysis. AUC of the best-performing model (CpG markers, 2-point fertility gap, LASSO) as a function of training set size (expressed as a percentage of the full training set). The training set was progressively reduced while the test set was kept identical across all runs. The black dots represent AUC in one resampling iteration. The blue dot represents the mean AUC over 20 resampling iterations.

**Figure S6.** Distribution of heritability estimates for CpG markers selected in the best-performing LASSO model (CpG markers, 2-point fertility gap), stratified by selection frequency across 20 resampling iterations: Singletons (selected once), Low (2–5 times), Medium (6–10 times), and High (11–20 times). The overall median heritability across all CpGs considered in the study (0.19) is shown for reference.
